# Chk1 Inhibitor MK-8776 Restores the Sensitivity of Chemotherapeutics in P-glycoprotein Overexpressing Cancer Cells

**DOI:** 10.3390/ijms20174095

**Published:** 2019-08-22

**Authors:** Qingbin Cui, Chao-Yun Cai, Jing-Quan Wang, Shuang Zhang, Pranav Gupta, Ning Ji, Yuqi Yang, Xingduo Dong, Dong-Hua Yang, Zhe-Sheng Chen

**Affiliations:** 1 School of Public Health, Guangzhou Medical University, Guangzhou 511436, China; 2 Department of Pharmaceutical Sciences, College of Pharmacy and Health Sciences, St. John’s University, Queens, NY 11439, USA

**Keywords:** P-gp, multidrug resistance, MK-8776, restore, sensitivity

## Abstract

P-glycoprotein (P-gp), which is encoded by the ATP-binding cassette (ABC) transporter subfamily B member 1 (*ABCB1*) gene, is one of the most pivotal ABC transporters that transport its substrates across the cell membrane. Its overexpression is one of the confirmed causes of multidrug resistance (MDR), which results in the failure of cancer treatment. Here, we report that checkpoint kinase (Chk) 1 inhibitor MK-8776, a drug candidate in clinical trial, can restore the sensitivity of chemotherapeutics that are substrates of P-gp in KB-C2, SW620/Ad300 cells and human embryonic kidney (HEK)293/*ABCB1* cells that overexpress P-gp. MK-8776 remarkably enhanced the cellular [^3^H]-paclitaxel accumulation and suppressed the efflux function of P-gp without reducing its expression and affecting its cellular localization in cancer cells. Furthermore, MK-8776 (0–40 μM) stimulated the activity of ATPase in P-gp, which was 4.1-fold greater than the control. In addition, MK-8776 formed a cation–π bond and π–π interaction with key residues of the substrate-binding site in P-gp, as indicated by computer-aided molecular docking study. Our study indicated that MK-8776 may significantly enhance the sensitivity of chemotherapeutics that are substrates of P-gp, providing important information for its application in the reversal of MDR.

## 1. Introduction

ATP-binding cassette (ABC) transporters present in the cell membrane are a group of ATP-dependent pumps that transport their substrates in and/or out of cells [1,2]. The substrates include endogenous inorganic anions, certain metal ions, amino acids and their formed peptides and sugars, as well as various hydrophobic compounds, exogenous chemicals, drugs and their cellular metabolites [2,3]. P-glycoprotein (P-gp), also named multidrug resistance 1 (MDR1), is encoded by the ABC subfamily B member 1 (*ABCB1*) gene. It is one of the most critical ABC transporters that localize in the cell membrane of various organs and the blood–brain barrier. Generally, P-gp mediates the resistance of cytotoxic and targeted chemotherapeutics by transporting them out of cancer cells without chemical modification [4]. Previous studies have found that P-gp is overexpressed in resistant breast cancer, lung cancer and colon cancer cells [5,6,7], as well as in patients with neuroblastoma, acute myeloid leukemia, acute lymphoblastic leukemia [8,9,10,11] and many other cancers [12], indicating that it is an important target, the inhibitor of which is emerging as one promising approach to overcome MDR [13,14]. However, the development of such small molecules has been unsuccessful, mainly due to unexpected severe adverse effects and/or low efficacy in improving the therapeutic outcomes as measured in clinical trials [15,16].

**Figure 1:**
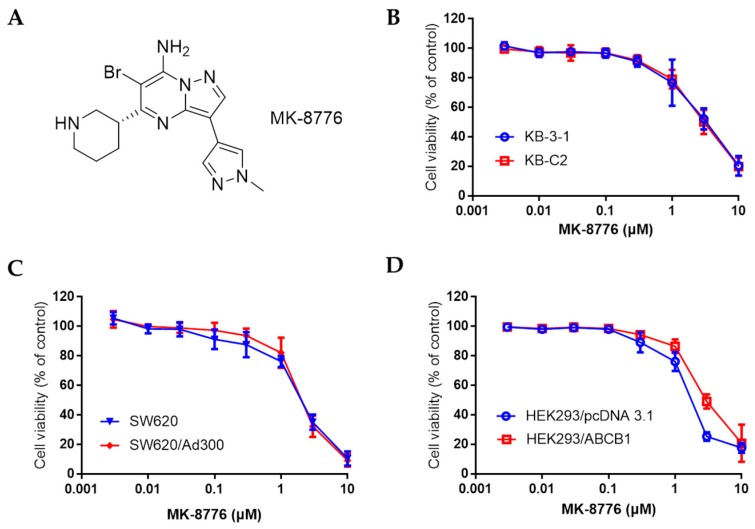
The chemical structure of MK-8776 (**A**), and the cell viability curve of human epidermoid carcinoma cell line KB-3-1 and its drug resistant KB-C2 cells (**B**), colon cancer cell line SW620 and doxorubicin-resistant SW620/Ad300 cells (**C**) that overexpress P-gp, HEK293/*pcDNA3.1* and *ABCB1* transfected HEK293/*ABCB1* cells (**D**) upon treatment with MK-8776.

Recently, novel molecules such as natural products and many tyrosine kinases inhibitors (TKIs) are found to be P-gp modulators [17,18]. The combination of certain TKIs at non-toxic concentration with chemotherapeutics can reverse MDR via the suppression of the expression or inhibition of the efflux function of P-gp [13,19]. Checkpoint kinases (Chk), composed of two subtypes, Chk1 and Chk2, are mediators that play crucial roles in regulating DNA damage and the cell cycle by phosphorylating their substrate proteins, leading to the activation of DNA damage checkpoints, DNA repair, cell cycle transition, and apoptosis inhibition [20,21]. Activated by the single-stranded DNA or double-strand breaks (DSBs) of DNA, Chks, which are phosphorylated by ataxia telangiectasia mutated (ATM) and Rad3 related kinase (ATR), can promote the G2/M-phase and intra-S-checkpoints (Chk1), or evoke the p53-associated G1/S-phase checkpoint and S-phase cell cycle arrest (Chk2) [22], which in turn provide the time for DNA repair before allowing the cells with damaged DNA to perform mitosis [23]. MK-8776 (Figure 1A) is a Chk1 inhibitor in clinical trials for certain advanced solid tumors and acute myeloid leukemia [24,25]. MK-8776 works synergistically with radiotherapy and certain chemotherapy to combat human non-small lung cancer (NSCLC), leukemia and triple negative breast cancer via DSB repair inhibition, cell cycle arrest and autophagy inhibition [26,27,28]. In this work, we report that MK-8876 may restore the sensitivity of chemotherapeutics that are P-gp substrates to cancer cells that overexpress P-gp, suggesting its potential in circumventing P-gp-mediated MDR in cancer.

## 2. Results

### 2.1. MK-8776 Restored the Sensitivity of Chemotherapeutics in P-gp-Overexpressing Cancer Cells

We first determined the non-toxic concentration of MK-8776 tested for its resensitizing effects by the 3-(4,5-dimethylthiazol-yl)-2,5-diphenyltetrazolium bromide (MTT) assay. As shown in Figure 1B–D, in KB-3-1, SW620, HEK293 and their corresponding drug-resistant KB-C2, SW620/Ad300, HEK293/*ABCB1* cells that overexpressed P-gp, the IC_50_ values of MK-8776 towards these cells ranged from 2–6 μM. Non-toxic concentration was at around 1 μM, so 0.3 and 1 μM were adopted for the re-sensitizing study.

Next, we tested the cytotoxicity of P-gp substrates, including doxorubicin, paclitaxel and colchicine, with or without co-administration of MK-8776. In this experiment, the positive control, verapamil (3 μM), a non-selective P-gp inhibitor, and negative control, cisplatin, a non-substrate of P-gp, were also measured.

As shown in Table 1 and Table 2, doxorubicin, paclitaxel and colchicine exhibited much higher sensitivity towards KB-3-1, HEK293 and SW620 cells than KB-C2, HEK293/*ABCB1* and SW620/Ad300 cells that overexpress P-gp. The resistance fold (RF, IC_50_ values of substrates in the resistant cell lines in the presence or absence of MK-8776 or verapamil divided the IC_50_ values of substrates in the parental cells without MK-8776 or verapamil) ranged from 97.88 to 695.75. The overexpression of P-gp indeed caused resistance properties for its substrates, as confirmed in HEK293/*ABCB1* cells (RF 10.34–51.46).

Importantly, when co-administrated with MK-8776, these chemotherapeutics demonstrated significantly lower IC_50_ values to KB-C2 and SW620/Ad300 cells compared with that in the absence of MK-8776. Similarly, MK-8776 restored the sensitivity of all the three chemotherapeutics to P-gp-transfected HEK293/*ABCB1* cells. In addition, the co-administration of MK-8776 showed no impact to KB-3-1, SW620, and HEK293 cells and no impacts on cisplatin in all the cell lines.

### 2.2. MK-8776 Increased P-gp Substrate [^3^H]-Paclitaxel Accumulation and Suppressed its Efflux in KB-C2 Cells

The efflux mediated by P-gp may severely restrain the intracellular accumulation of certain chemotherapeutics, leading to drug resistance [13]. As MK-8776 restored the sensitivity of P-gp substrates, we further measured its effects on P-gp efflux function by evaluating the intracellular accumulation and extracellular concentration of radioactive [^3^H]-paclitaxel at different times. The P-gp-overexpressing KB-C2 cells were treated with or without MK-8776 (0.3, 1 μM) for 2 h, and then the intracellular concentration of [^3^H]-paclitaxel was measured by Packard TRI-CARB 1900CA liquid scintillation analyzer. Moreover, the extracellularity of [^3^H]-paclitaxel was also measured.

**Figure 2:**
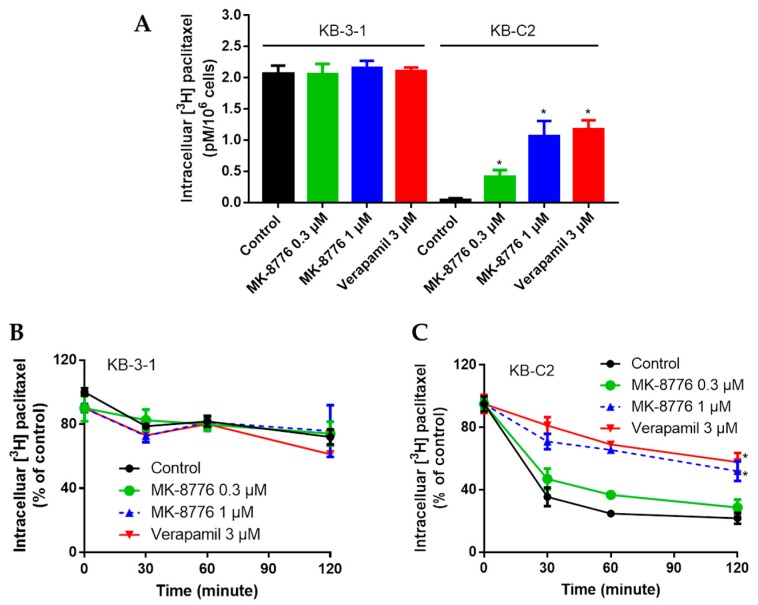
Effects of MK-8776 on the intracellular accumulation of [^3^H]-paclitaxel in KB-C2 cells that overexpress P-gp (**A**,**C**) and their parent KB-3-1 cells (**A**,**B**). * *p* < 0.05 vs. control.

As shown in Figure 2, in KB-C-2 cells, the [^3^H]-paclitaxel concentration decreased significantly and [^3^H]-paclitaxel efflux increased significantly compared with that in their parental KB-3-1 cells. Pretreatment with MK-8776 significantly increased the accumulation and inhibited the efflux of [^3^H]-paclitaxel in KB-C2 cells, while MK-87776 showed no such effects on KB-3-1 cells. These results indicated that MK-8776 may impact the efflux function of P-gp.

### 2.3. MK-8776 Did Not Alter the Expression of P-gp in KB-C2 Cells

We then tested the expression of P-gp on MK-8776 in KB-C2 cells. Cells were treated with MK-8776 with different times (1 μM for 0, 24, 48, 72 h) and doses (0.1, 0.3, 1 μM for 72 h). Then, the P-gp expression in different groups was measured by Western blot assay. KB-3-1 cells were used as a negative control.

**Figure 3:**
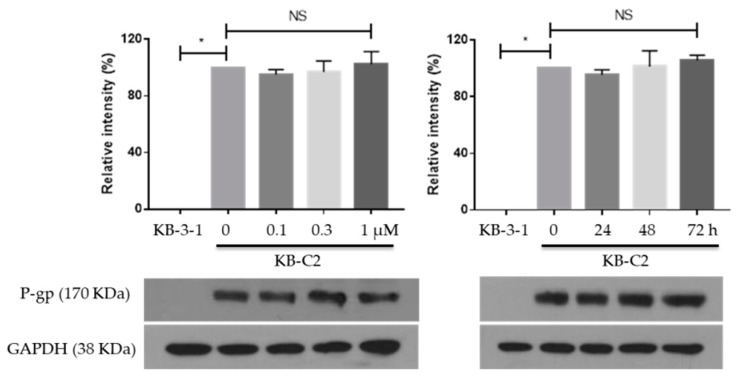
Effects of MK-8776 on P-gp expression in KB-C2 cells. * *p* < 0.05 vs. control. NS, not significant.

The results in Figure 3 show that KB-3-1 cells expressed no P-gp, but KB-C2 cells expressed high P-gp, which could lead to the MDR property. However, P-gp expression was not significantly altered by MK-8776. The above two results indicated the MK-8776 might suppress the efflux function without altering the cellular expression of P-gp.

### 2.4. MK-8776 Did Not Alter P-gp Subcellular Localization

Since MK-8776 did not alter the P-gp expression in cells, we next tested the cellular localization of P-gp by immunofluorescence assay. SW620/Ad300 cells were treated with 1 μM of MK-8776 for 0, 24, 48, 72 h, and then P-gp was labeled by Alexa Fluor 488 dye. In this experiment, SW620 cells were used as a negative control, and DAPI was used to counterstain the nuclei.

**Figure 4:**
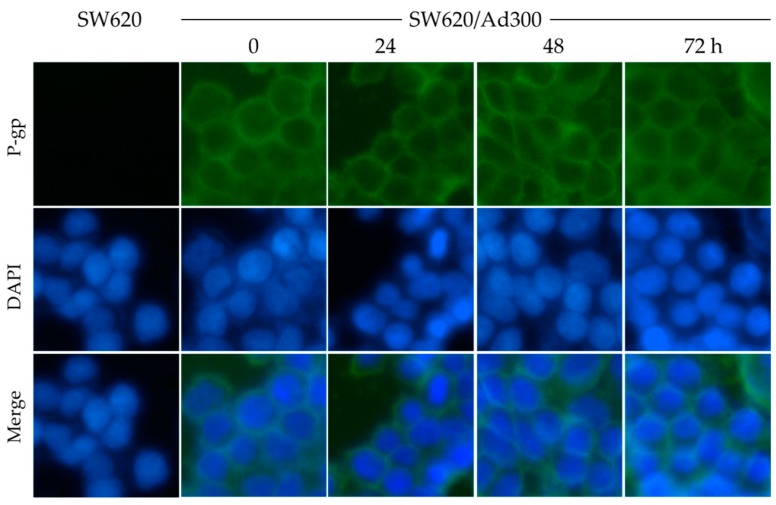
Effects of MK-8776 on the cellular localization of P-gp in P-gp-overexpressing SW620/Ad300 cells. Pictures were taken under 10× magnification microscope. Green: P-gp. Blue: nuclei.

The results in Figure 4 indicate that P-gp accumulated mostly in the membrane of cancer cells, while the MK-8776 treatment did not alter the cellular localization of P-gp significantly.

### 2.5. MK-8776 Stimulated ATPase Activity

**Figure 5:**
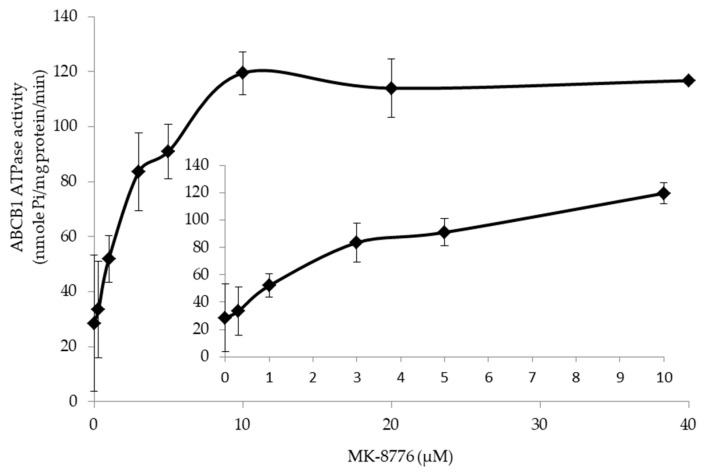
MK-8776 stimulated the ATPase of P-gp.

To obtain more details of the action mode of MK-8776 on P-gp, we determined the ATPase upon MK-8776 (0–40 μM) by measuring the P-gp-mediated ATP hydrolysis. The result in Figure 5 shows that stimulated ATPase activity of P-gp in a dose-dependent manner was observed upon MK-8776. MK-8776 exerted a concentration of 50% stimulation at 3.8 μM and exhibited 4.1-fold greater stimulation than that of the control.

### 2.6. Induced-Fit Docking (IFD) Simulation Interactions between P-gp and MK-8776

**Figure 6:**
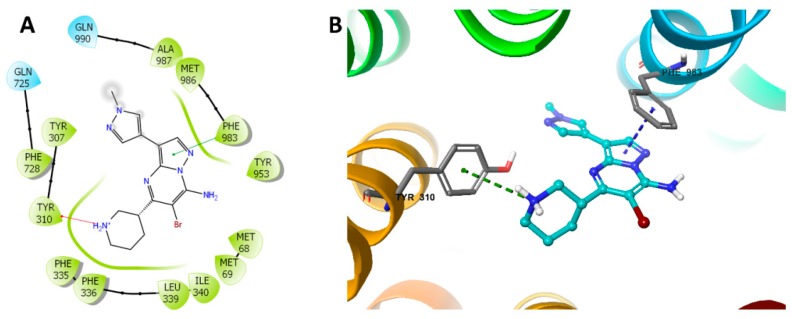
Docking study of MK-8776 with human P-gp. (**A**) The generated 2D figure of the docked position of MK-8776 within the P-gp crystal. The polar residues are indicated with cyan bubbles, and the hydrophobic residues are indicated with green bubbles. Furthermore, the cation–π bond in the figure is shown as red arrow, and the π–π interaction is shown as green line. (**B**) The enlarged 3D figure of the docked position of MK-8776 within the drug-binding site of human P-gp. The structure of MK-8776 is shown as a ball and stick mode with the atoms colored as follows: carbon with cyan, nitrogen with blue, bromine with dark red. The important residues of P-gp are shown as sticks, with the atoms colored as follows: carbon with grey, nitrogen with blue, oxygen with red, hydrogen with white. The cation–π bond is indicated with green dotted line. The π–π stacking interactions are indicated with a blue dotted line.

We performed the IFD to evaluate the interaction between MK-8776 and P-gp by molecular docking analysis. Our results showed that the docking score of of MK-8776 to P-gp was −7.535 kcal/mol, indicating a good affinity. Figure 6 showed that there was one cation–π bond between the ionized amine of MK-8776 and the Tyr310 of human P-gp. The imidazolopyrimidine group of MK-8776 formed a π–π interaction with Phe983 of residues of P-gp in the drug-binding site.

## 3. Discussion

MDR, which is one of the leading causes of treatment failure in cancer, refers to a phenomenon whereby, after a short time of chemotherapeutic treatment, cancer cells become resistant to other chemotherapeutics with different mechanisms [29]. A very limited number of drugs are available for patients with late-term cancer or recurrent cancer, which become resistant to multiple therapeutic modalities. Various mechanisms may lead to MDR, such as the increase of drug efflux, the activation of cell signaling, growth, migration and other defense systems, the inhibition of apoptosis, and activation of DNA repair [30,31,32,33]. Among all these mechanisms, the overexpression of the ABC transporters which regulate the uptake and efflux of chemotherapeutics [15,16], especially P-gp, is one of the recognized pathways that confers MDR to small molecule-based therapy [34,35]. Beside the development of special P-gp regulators, drug repurposing has emerged as another appealing strategy as those molecules have been assessed in clinical/preclinical trials and considered as safe enough [36,37]. Many small molecular drugs, including TKIs [38], phosphodiesterase inhibitors [39], and fluoroquinolone antibiotics [40], antagonize P-gp mediated MDR at non-cytotoxic concentrations. These studies provide important information for future clinical trials and for further new drugs targeting P-gp.

MK-8776 is a Chk1 inhibitor that is under clinical trials for acute leukemia and certain advanced solid tumors. In a phase-I clinical trial, MK-8776 was shown to be well tolerated by single or combination use, accompanied with positive outcomes of clinical efficacy [25]. The results of phase II are yet to be announced. A previous study found that, beside the mechanism of Chk-related DNA repair and cell cycle suppression that can sensitize radiotherapy, or gemcitabine, which induces DNA DSBs [27,28], MK-8776 could also exert its re-sensitizing effects to different therapies via autophagy inhibition [26].

In this current work, we found that MK-8776 could restore the sensitivity of chemotherapeutics which are P-gp substrates to P-gp-overexpressing cancer cells by regulating the efflux function. The chemotherapeutics that are P-gp substrates exhibited resistance profiles (up to more than 600-fold) to KB-C2 and SW620/Ad300 cells but not to KB-3-1 and SW620 cells which expressed no P-gp. The combination of MK-8776 at 0.3 and 1 μM (non-toxic concentration) remarkably lower the IC_50_ values of KB-C2 and SW620/Ad300 cells, suggesting its re-sensitizing effects. MK-8776 exhibited similar effects to these three drugs in HEK293/*ABCB1* cells, which are normal cell lines that were transfected with *ABCB1*; meanwhile, it exhibited no such effects to the non-Pgp substrate cisplatin, indicating its effects in modulating P-gp functions. As we can observe, since all cells expressed a high level of P-gp [40], the RF of the chemotherapeutics and the reversal effects by MK-8776 in KB-C2 or SW620/Ad300 cells and HEK293/*ABCB1* cells were different. The RF in KB-C2 or SW620/Ad300 cells were higher than in HEK293/*ABCB1* cells, and the reversal effects of MK-8776 and verapamil in KB-C2 or SW620/Ad300 cells were weaker than in HEK293/*ABCB1* cells. This is probably due to the other resistant factors (except the overexpression of P-gp) of different chemotherapeutics on different cancer cells, and/or different levels of P-gp, suggesting that higher transporter expression levels may require a higher inhibitor concentration to achieve a certain inhibitory effect [41,42].

To explore the mechanism, we further conducted the Western blot and immunofluorescence study to test the effects of MK-8776 to the expression and cellular localizations of P-gp. The results indicated that MK-8776 did not alter either of them, while it could stimulate the ATPase of P-gp, an effect that was similar to the P-gp inhibitor verapamil [43]. This effect of stimulating ATPase may competitively limit the uptake of substrate of P-gp, which can lead to the inhibition efflux function. Indeed, MK-8776 significantly suppressed the efflux of [^3^H]-paclitaxel, resulting in more accumulation of [^3^H]-paclitaxel in KB-C2 cells. In addition, the molecular modeling indicated that MK-8776 could form a cation–π bond and π–π interaction with the drug-binding pocket of human P-gp, which may lead to the inhibition of efflux. Our work provided useful information regarding the potential application of MK-8776 in the treatment of resistant cancer that overexpresses P-gp.

Although the discovery of P-gp inhibitors so far seems not to have been successful, the full functions of P-gp in drug transport and diseases progression need to be revealed [15]. The findings of our group appear to suggest that rather than being tyrosine kinase-related (although this could not be excluded), those agents that show ABC transporter-regulating effects are probably structure-related, suggesting that certain structures may tend to regulate the efflux functions of ABC transporters. Their further application in clinic practice could be achieved after their safety/pharmacokinetics profiles are combined with chemotherapeutics, and the exact mechanism and binding sites have been fully deciphered.

## 4. Materials and Methods

### 4.1. Chemicals and Reagents

Chk1 inhibitor MK-8776 was a kind gift from ChemieTek (Indianapolis, IN, USA). Dulbecco’s modified Eagle’s Medium (DMEM), fetal bovine serum (FBS), bovine serum albumin (BSA), antibacterial penicillin/streptomycin solution (100×), trypsin (0.25%) were purchased from Hyclone (GE Healthcare Life Science, Pittsburgh, PA, USA). Monoclonal antibodies, P-gp (C219) and Glyceraldehyde-3-Phosphate Dehydrogenase (GAPDH, MA5-15738), and secondary antibody Alexa Fluor 488 conjugated goat anti-mouse IgG used in immunofluorescence assay were purchased from Thermo Fisher Scientific Inc (Rockford, IL, USA), Triton X-100, 3-(4,5-dimethylthiazol-yl)-2,5-diphenyltetrazolium bromide (MTT), propidium iodide (PI), 36% paraformaldehyde, chemotherapeutics paclitaxel, doxorubicin, colchicine, cisplatin and verapamil were purchased from Sigma-Aldrich (St. Louis, MO, USA). Radioactive [^3^H]-paclitaxel (15 Ci/mmol) was purchased from Moravek Biochemicals, Inc (Brea, CA, USA). The chemical materials for the ATPase assay were the same as those in our previous work [14,19].

### 4.2. Cell Lines and Cell Culture

Human epidermoid carcinoma KB-3-1 cells and their colchicine-selected KB-C2 cells that overexpress P-gp, colon cancer cells SW620 and doxorubicin-resistant SW620/Ad300 cells that overexpress P-gp [44], human embryonic kidney 293 (HEK293) cells transfected with the empty *pcDNA3.1* vector, HEK293/*pcDNA3.1* cells, and HEK293 cells transfected with the vector containing a full length of *ABCB1* and HEK293/*ABCB1* cells were used for the chemotherapeutic re-sensitizing study. HEK293/pcDNA3.1 and HEK293/*ABCB1* cells were cultured in a medium containing 2 mg/mL of G418 for two weeks before re-sensitizing assay. SW620 and SW620/Ad300 cells were used for the immunofluorescence analysis. All cells were cultured in DMEM that contained 10 FBS and 1% antibacterial at 37 °C, 5% CO_2_. All the drug-resistant cell lines were allowed to grow as an adherent monolayer in a drug-free culture media for more than 2 weeks before the study.

### 4.3. Cytotoxicity experiments

The cytotoxicity and re-sensitizing experiments of MK-8776 to KB-3-1, KB-C2, SW620, SW620/Ad300, HEK293/pcDNA3.1, HEK293/*ABCB1* cells were determined by the MTT colorimetric assay as previously reported [45]. MK-8776 at 0.3 µM and 1 µM were applied based on the results of cytotoxicity experiments at non-cytotoxic concentrations. All experiments were repeated at least three times in triplicate, and the mean and standard deviation (SD) values were calculated by GraphPad Prism 7.00 software (GraphPad Software Inc., La Jolla, San Jose, CA, USA). The non-selective P-gp inhibitor verapamil (3 μM) was used as the positive control. Non-P-gp substrate cisplatin was used as the negative control drug.

### 4.4. Western Blot Analysis

The P-gp expression of KB-C2 cells upon MK-8776 (both dose-dependent—0, 0.1, 0.3, 1 µM—and time-dependent—0, 24, 48, 72 h) were determined by sodium dodecyl sulfate polyacrylamide gel electrophoresis (SDS-PAGE). Generally, equal amounts of cell lysates (30 µg protein) were resolved by SDS-PAGE and electrophoretically transferred onto polyvinylidene fluoride membranes. The presence of P-gp was confirmed using monoclonal P-gp antibody C219 (dilution 1:1000 in 5% milk in PBS). GAPDH (dilution 1:1000 in 5% milk in PBS) was used as a load control. The band quantification was conducted by Image J software (NIH, MD, USA). The detailed protocol was carried out according to our previous work [46].

### 4.5. Immunofluorescence Analysis

Immunofluorescence analysis was applied to study the impact of MK-8776 to the distribution of P-gp. SW620 and SW620/Ad300 cells were seeded in 24-well plates (1 × 10^4^/well) and cultured for 24 h, followed by incubation with 1 µM MK-8776 for 0, 24, 48 and 72 h, respectively. Then, after being washed with PBS (3 times), the cells were fixed (4% paraformaldehyde, 5 min) and permeabilized (0.1% Triton X-100 in PBS, 5 min) before being blocked with 6% BSA for 1 h at 37 °C. The presence of P-gp was confirmed by monoclonal ABCB1 antibody C219 (dilution 1:1000 in 6% BSA in PBS) for incubation at 4 °C overnight. Secondary antibody Alexa Fluor 488 (dilution 1:1000 in 6% BSA in PBS) was used for incubation at 37 °C for 1 h. After washing with cold PBS (3 times), DAPI (1 μg/mL in PBS) was used to counterstain the nuclei. Immunofluorescence images were collected using an EVOS FL Auto fluorescence microscope (Life Technologies Corporation, Gaithersburg, MD, USA).

### 4.6. ATPase Assay

The ATPase activity of P-gp upon MK-8776 (0–40 μM) was tested by the PREDEASY ATPase Kits (TEBU-BIO nv, Boechout, Belgium) according to manufacturer’s instructions [46].

### 4.7. [^3^H]-Paclitaxel Accumulation and Efflux Assay

P-gp substrate and radioactive [^3^H]-paclitaxel was applied to measure the accumulation and the efflux effects of P-gp upon MK-8776 (0.3, 1 μM) by examining its intracellular and extracellular concentrations at different time. The non-selective P-gp inhibitor verapamil (3 μM) was used as the positive control [47].

### 4.8. Molecular Modeling of Human ABCB1 Homology Model

We conducted a computer-aided molecular docking study to predict the binding details of MK-8776 to P-gp. The docking experiments were performed according to the reported protocols [19,48] with Schrodinger 2018-1 software (Schrödinger, Cambridge, MA, USA). The ligand was essentially prepared by the default condition. Human ABCB1 (PDB: 6QEX) protein was prepared [49] and the docking grid was generated. Glide XP docking was conducted and the induced-fit docking (IFD) was performed after the receptor grid was generated by selecting residues in the substrate-binding site of P-gp.

### 4.9. Statistical Analysis

IC50 values and protein quantities were exhibited as the mean ± SD. All experiments were repeated at least three times, and the data were analyzed by a one-way or two-way ANOVA by GraphPad Prism 7.00 software. *p* < 0.05 was considered as significantly different.

## 5. Conclusions

The Chk inhibitor MK-8776 restored the sensitivity of chemotherapeutics that were P-gp substrates to P-gp-overexpressing cancer cell by suppressing the efflux.

## Figures and Tables

**Table 1:** MK-8776-sensitized doxorubicin, paclitaxel, and colchicine in KB-C2 and HEK293/*ABCB1* cells.

Compounds	IC_50_ ± SD ^a^ (μM) (RF ^b^)			
	KB-3-1	KB-C2	HEK293/*pcDNA3.1*	HEK293/*ABCB1*
Doxorubicin	0.017 ± 0.011 (1.00)	1.664 ± 0.064 (97.88)	0.061 ± 0.020 (1.00)	0.631 ± 0.150 (10.34)
+ MK-8776 (0.3 μM)	0.015 ± 0.003 (0.88)	0.470 ± 0.041 (27.64) *	0.067 ± 0.017 (1.10)	0.181 ± 0.014 (2.97) *
+ MK-8776 (1 μM)	0.014 ± 0.004 (0.82)	0.013 ± 0.004 (0.76) *	0.058 ± 0.027 (0.95)	0.056 ± 0.033 (0.92) *
+ Verapmil (3 μM)	0.014 ± 0.006 (0.82)	0.016 ± 0.005 (0.94) *	0.061 ± 0.008 (1.00)	0.084 ± 0.009 (1.38) *
Paclitaxel	0.004 ± 0.002 (1.00)	2.783 ± 0.053 (695.75)	0.073 ± 0.027 (1.00)	3.757 ± 0.312 (51.46)
+ MK-8776 (0.3 μM)	0.004 ± 0.001 (1.00)	0.144 ± 0.013 (36.00) *	0.122 ± 0.050 (1.67)	0.255 ± 0.084 (3.49) *
+ MK-8776 (1 μM)	0.003 ± 0.001 (0.75)	0.092 ± 0.004 (23.00) *	0.100 ± 0.020 (1.37)	0.047 ± 0.004 (0.64) *
+ Verapamil (3 μM)	0.003 ± 0.001 (0.75)	0.017 ± 0.002 (4.25) *	0.068 ± 0.003 (0.95)	0.094 ± 0.003 (1.9) *
Colchicine	0.012 ± 0.004 (1.00)	7.732 ± 0.240 (644.33)	0.066 ± 0.001 (1.00)	1.538 ± 0.090 (23.30)
+ MK-8776 (0.3 μM)	0.009 ± 0.005 (0.75)	0.292 ± 0.203 (24.33) *	0.058 ± 0.007 (0.88)	0.126 ± 0.106 (1.91) *
+ MK-8776 (1 μM)	0.009 ± 0.003 (0.75)	0.009 ± 0.030 (0.75) *	0.048 ± 0.009 (0.73)	0.047 ± 0.021 (0.71) *
+ Verapamil (3 μM)	0.008 ± 0.003 (0.66)	0.006 ± 0.015 (0.5) *	0.056 ± 0.006 (0.85)	0.050 ± 0.008 (0.76) *
Cisplatin	2.508 ± 0.432 (1.00)	3.027 ± 0.343 (1.21)	2.660 ± 0.430 (1.00)	3.336 ± 0.900 (1.25)
+ MK-8776 (0.3 μM)	2.440 ± 0.264 (0.97)	2.770 ± 0.167 (1.10)	2.378 ± 0.136 (0.89)	2.727 ± 0.592 (1.03)
+ MK-8776 (1 μM)	2.431 ± 0.179 (0.97)	2.652 ± 0.087 (1.05)	2.474 ± 0.286 (0.93)	2.611 ± 0.353 (0.98)
+ Verapamil (3 μM)	2.309 ± 0.641 (0.92)	2.098 ± 0.230 (0.84)	2.388 ± 0.452 (0.90)	3.115 ± 0.433 (1.17)

* *p* < 0.05 vs. control. ^a^ Three independent experiments which were performed in triplicate. ^b^ IC_50_ values of substrates in the resistant cell lines in the presence or absence of MK-8776 or verapamil divided by the IC_50_ values of substrates in the parental cells without MK-8776 or verapamil.

**Table 2:** MK-8776-sensitized doxorubicin and paclitaxel in SW620/Ad300 cells.

Compounds	IC_50_ ± SD ^a^ (μM) (RF ^b^)	
	SW620	SW620/Ad300
Doxorubicin	0.031 ± 0.014 (1.00)	9.950 ± 2.023 (320.97)
+ MK-8776 (0.3 μM)	0.028 ± 0.016 (0.90)	1.362 ± 0.122 (43.94) *
+ MK-8776 (1 μM)	0.035 ± 0.012 (1.13)	0.426 ± 0.184 (13.74) *
+ Verapmil (3 μM)	0.038 ± 0.021 (1.23)	0.096 ± 0.023 (3.10) *
Paclitaxel	0.091 ± 0.015 (1.00)	21.19 ± 6.25 (232.86)
+ MK-8776 (0.3 μM)	0.076 ± 0.038 (0.84)	1.784 ± 0.125 (19.60) *
+ MK-8776 (1 μM)	0.074 ± 0.003 (0.81)	0.597 ± 0.566 (6.56) *
+ Verapamil (3 μM)	0.113 ± 0.006 (1.24)	0.639 ± 0.023 (7.03) *
Cisplatin	1.481 ± 0.676 (1.00)	1.514 ± 0.398 (1.02)
+ MK-8776 (0.3 μM)	1.329 ± 0.156 (0.90)	1.423 ± 0.438 (0.94)
+ MK-8776 (1 μM)	1.228 ± 0.181(0.83)	1.266 ± 0.295 (0.84)
+ Verapamil (3 μM)	1.164 ± 0.107 (0.79)	1.851 ± 0.364 (1.25)

* *p* < 0.05 vs. control. ^a^ Three independent experiments that were performed in triplicate. ^b^ IC_50_ values of substrates in the resistant cell lines in the presence or absence of MK-8776 or verapamil divided by the IC_50_ values of substrates in the parental cells without MK-8776 or verapamil.
